# Reasonable Talk on the Subject of Filling Teeth

**Published:** 1872-05

**Authors:** H. Scott

**Affiliations:** Lancaster, O.


### Reasonable Talk on the Subject of Filling Teeth.

BY H. SCOTT, LANCASTER, O.

I hope I shall not be voted obtrusive for again offering some suggestions on tooth filling. I am aware that the subject has been talked over a great deal; but dentists will not tire of it. It is a matter of superior importance, especially in view of the conflictions everywhere met with as we hear each man talking from his own standpoint. The various views presented by really good men cannot fail, as I believe, to confuse more or less those just starting out on their own account, as well as some who have been at work for many years, but who are not always satisfied, it may be, with their own methods. Thus we have estimations in regard to how heavy the mallet should be, varying from one to ten ounces; as also on the relative merits of the light and heavy foils; running from number four up to number one hundred and sixty, and even higher I believe; as well as on the adaptation of the smooth or serrated points, and soft and adhesive foils, etc. I do not propose to enter into any of these controversies. I have nothing to say in this connection about the weight or material of mallets; the numbers of foils; or the kinds of points to be used; assuming that a workman will accomplish success with any of the ordinary methods or implements in common use, and with any number of good foil. Before passing on, however, I have a single suggestion to make, viz: that those who are succeeding well should shift their base of manipulations with caution, and by slow movements to avoid the inevitable blunders always consequent upon taking a new departure. But I by no means intend to say that operators are to neglect to avail themselves of valuable suggestions by our pioneers; only touch them cautiously at first—that is all.

The lessons in the above conflicting theories briefly hinted at are, that absolute perfection in this highest and most responsible function of our profession has not yet been attained. But in going on to perfection, how are we to proceed? Not merely by the continual attempted adoption of every claimed better method, at least not until such method has been wisely philosophised, and its superior claims clearly perceived. The intentions of successful filling are to be kept constantly in view and the means made subservient to them. First, the nerve membrane must not be exposed; second, the cavity must be anchored, and firm smooth borders secured; and thirdly, the metal used so accurately fitted and made firm that no moisture can by possibility ever reach the dentine. This is all that dentists can do; and it is enough.

In making my "reasonable talk," I wish to refer to Professor Wright's paper, published in THE REGISTER for October, 1871. I read his article with a great deal of interest, and judged his views to be most reasonable and common sense; and after the matter was referred to in the meeting at Columbus, on the 7th of December last, disapprovingly by many of the prominent dentists present, as I thought, I referred to it again, and have no reasons for changing my opinion. I will give several reasons why I think his logic just, and his strictures reasonable.

If I understood the professor, and I think I did, unless he possessed peculiar art in disguising himself, he wanted none but good work done. His dissertation seemed to be about the tedious, and expensive, and complicated methods of doing the work, where equal success could be reached easier and quicker. In much that he said, therefore, I agree with him. The art of filling teeth is capable of being made a blessing to mankind; and may be placed also within the reach of those of even moderate means, who would be able to pay reasonable, and still remunerative fees to the dentist; but who, by the rule of high prices that must be charged for the more tedious and finer manipulations of the coffer-dam system generally, and the fine jewel polish, would ever be able to to have even one tooth filled? If therefore the practice of tooth-filling should be ruled and held to the fine work only (not to speak of good), then the few alone who are favored with ample means could avail hemselves of its benefits. A dentist's time is his capital, and he can't work two to five hours on a single filling, and find his own gold, for a dollar and a half or two dollars. This is my first presentation of the subject.

But it may be asked "would you suggest different grades of work in quality, to correspond with the prices paid, or according to the purse of the patient?" That is exactly what I do not mean to say; and here the question will turn on the inquiry, whether good gold fillings can be made that will endure and save the teeth permanently for $1.50 or $2.00 each. I affirm that they can, in small and ordinary cavities. And I affirm too that they can be made of soft foils without the application of the rubber dam, and by hand force at that, though I use the mallet constantly now, and never fill a tooth without it. I do not say that men should fill teeth with gold for $1.50 or $2.00, but I do say it can be done where the cavities are small and without complications. But even the country practitioner will be justifiable in charging higher rates for large fillings, according to the time and material required, which patients readily understand and appreciate.

Until with the last ten or fifteen years I did the most of my filling with hand force; and some of the most durable work I have done during my thirty-three years' practice was made in that way. Two days ago a lady called at my office with twelve gold fillings which she said I had made for her sixteen years before. They were every one in place just as I left them, the margins all perfect, and not the slightest indication of decay at any point. Eight of them were in the incisors, two to each tooth; one in each cuspid, and the other two in the crown surfaces of the first molars, all of the upper jaw.

In contrast with this case was that of a gentleman who had his central incisors built out in contour two years ago, and for which he paid $18.00. I found that the fillings did not fit the margins very accuratcly and that the tooth substance had crumbled all around the gold. One of the fillings was dangling by its anchorage. I removed the fillings as the only thing that could be done, and will engraft pivot-crowns for the present. Now I ask all reasonable dentists if these two incisors were worth filling at all. when first seen, if they could not have been filled with soft foil for, say $3.00 each, with better chance for their remaining much longer, provided of course that the work had been accurately fitted to the well-prepared borders of the cavities. What return did this man get for his $18.00, in the two fine pieces of polished gold he had. But this was only one of many similiar cases I have seen. The teeth, I understand were necrosed at the time, they were filled; but the crowns were nearly all remaining, and ought to have been kept there at least a dozen years longer.

Of course every dentist understands that there are two principal causes of failure in tooth-filling; first, error in judgment as to when and what is to be done; and secondly, imperfection in doing it. A young man has just been in my office who had an upper incisor filled four weeks ago. His face was frightfully swollen, and he had not slept for a couple of nights. The tooth was loose in its socket, and I removed it at once, and upon examination found that the nerve membrane had been impinged from the first.

One of the most frequent causes of failure I have observed to be the unequal manner in which the foils are consolidated; and this, without skillful care in conducting the process, of filling, will occur more frequently under the mallet than hand force. If it be the aim of the operator to drive the foil together as compactly as it is capable of, the utmost care is necessary from the first blow of the mallet to the last, and with no more malleting on the surface than has been used throughout the process, lest the mass be contracted in some direction. Many errors of this kind are constantly committed. I have seen gold fillings so firmly built as to require a sharp drill to penetrate them, while at the same time at one or more points around them the probe could be easily passed down into the tooth substance. I recently asked a dentist if he used the mallet. He said he did, but never till he had his cavity filled, and then used it on the surface only. What does any one think would be the condition of a plug after the face of a hand filling had been malleted? And this man had been operating one-fifth part of a century, at least.

All honor is due to the pioneers of our profession for their untiring labors to perfect tooth filling, and I shall always be among the first to accord it to them. There is nothing in my remarks that is to be tortured into objections to the rubber dam, or the mallet, or the protracted labor of putting on fine finishes; on the contrary I am the advocate of everything that tends to exalt and make honorable the dental art. The question I present is, shall all dentists be turned into fine jewelry workers, expending time and labor that cannot be compensated but by the payment of high fees—fees altogether too high to be paid by any besides those of ample means and large incomes. Suppose a person in moderate circumstances has his teeth filled with tin or amalgam at about from one to two dollars a cavity, or gold at from one and a half to two dollars a cavity for ordinary sized cavities; and suppose the work to be so well done in every respect that decay is entirely arrested, and that the fillings remain intact, thus saving the teeth and making them permanently useful, I ask has not the ends of tooth filling been consumated? And what more is required? But if a very *nice* operation be made, and some of the essential requirements of *good* and successful work be overlooked, and yet a high price has been paid for such fine work, what advantage has there been secured? One man's fancy and purse may determine him to pay fifteen dollars for a fine pair of sewed French calf boots, while another can spare but six dollars for a pair of substantial kip that will wear longer than three pair of the former. Which would be the most judicious expenditure of money? And tooth filling is capable of being put upon the same footing. Or would any one say that no man should furnish other than the finest quality of boots, and that men should not be adjudged worthy of putting them on until they were able to plank down the fifteen dollars? Are the functions of boot-makers and dentists to be restricted to fine and fancy work by law or the rule of society, in order that boot-making and dentistry shall acquire the reputation of fine arts, at the expense, it may be, of utility? I shall insist that dentists are morally bound to extend the benefits of tooth-filling to all classes, by placing their charges within the reach of the workingman and the sewing girl, but never at the expense of good, and durable, and successful work. Beyond this let those who wish crowns built of gold, or large contour fillings, with exquisite finish, and where means are ample, avail themselves of such fine and high-priced work; it is all right and well, and let dentists whose fancy runs in that line do the work, and select their cases if they are better pleased in that way. But the poor and moderate in circumstances must not be cut off by a rate of charges altogether beyond their possibilities, because dentists are held to a code that confers the honors only on fine work and high fees. It is as if the physicians of a city or community bind themselves in a society to charge ten dollars, and no less, for an accouchment, with no consideration at all as to the ability of people to pay equally. None can fail to see what the consequences of such a rule would be; either the poor must do without the *accoucheur*, or get his services and never pay for them. There are those who could pay two or three dollars, but who could not pay ten.

If all the requirements of tooth-filling have been met, wanting only the surface polish, that may be omitted with no detriment at all, finishing simply with the file and burnisher, especially on all crown surfaces. I have passed thousands of such off as the mallet left them, barely cutting the metal to a smooth level with the enamel. And I have filled many hundreds of cavities in the wet, twenty and thirty years ago, that are as good to-day as any of the wants of successful tooth-filling require them to be. For wet work I want tough, soft foils, which I manipulate with the fingers into pellets and blocks corresponding with the size of the cavity to be filled; and after heating them in the flame, mallet them in as large pellets as can be started through the aperture. Of course I never attempt this method in any other than round cavities well-anchored. With tin foil I want dry cavities, deeming it far more important than with gold, where the wedging process is to be relied upon. But I never work in the wet when I can have the dry without the dam, which is easier in most mouths than many have been led to believe. When adhesive building up of gold is required, we can not proceed without the coffer dam, in cases requiring much time, and should never make the attempt. In making amalgam fillings I observe the same care in preparing cavities that is required for foils, and then depend for success on a great deal of firm packing, especially during the process of setting.

Thus have I tried to talk reasonably about tooth-plugging, and to simplify and put it on a rational basis. I hope I will be understood, and not misunderstood.

